# Semaglutide and beyond: a turning point in obesity pharmacotherapy

**DOI:** 10.1016/j.lanepe.2024.100860

**Published:** 2024-02-01

**Authors:** The Lancet Regional Health – Europe

The landscape of pharmacological interventions in combating obesity has long struggled with the formidable challenge of balancing efficacy and safety, with weight-loss drugs often falling short on both fronts. In this context, semaglutide, a glucagon-like peptide-1 (GLP-1) receptor agonist, initially developed for treating type 2 diabetes (marketed as Ozempic), has emerged as a notable breakthrough drug for obesity treatment (under the name Wegovy). Its efficacy in achieving non-surgical weight loss of 15% or more in adults and adolescents with overweight and obesity set a new standard previously unachievable without surgery. Beyond weight reduction, semaglutide also alleviates heart failure symptoms and reduces the risk of heart attack and stroke in individuals with obesity, presenting a promising intervention in the complex landscape of obesity pharmacotherapy.

Ongoing clinical trials are testing semaglutide as a potential treatment for a spectrum of health issues, including kidney and heart failure, addiction, Alzheimer's disease, Parkinson's disease, and metabolic dysfunction-associated steatohepatitis both in patients with and without obesity. If the trials show successful outcomes, semaglutide could be positioned as a compelling drug capable of addressing diverse conditions beyond type 2 diabetes and obesity. This could be similar to the beneficial effects achieved by statins beyond lipid lowering.

Earlier concerns regarding semaglutide and potential links to the development of certain cancers, particularly thyroid and pancreatic cancer, and associations with suicidal ideation, have now subsided. Recent data show no increased risk of developing any type of cancer and no association with suicidal ideation. In fact, there is even a suggestion of a reduced risk of suicidal ideation in individuals undergoing semaglutide treatment, although these findings are based on limited follow-up. While conclusive answers await future studies based on real-world data and long-term follow-up, the current reassuring results highlight the favourable safety profile.

The impressive treatment outcomes observed among people given semaglutide have fuelled a surge in demand for weight-loss drugs, particularly GLP-1 receptor agonists, also driven by endorsements from celebrities and influencers. Shortages in the supply of these drugs are anticipated to persist until the end of 2024. The heightened demand propelled Novo Nordisk, the manufacturer of semaglutide, to become Europe's most valuable company in 2023, surpassing the gross domestic product of its home country, Denmark.

In 2024, the availability of two highly promising drugs is set to further enhance obesity treatment with increased efficacy and improved user convenience. Tirzepatide, a dual GLP-1 and glucose-dependent insulinotropic polypeptide (GIP) receptor agonist, gained approval from the US Food and Drug Administration in November, 2023, surpassing semaglutide in terms of weight loss efficacy, with a 20.9% reduction in baseline weight. Additionally, an oral version of semaglutide has been developed to enhance treatment uptake and adherence. As novel, safe, and effective drugs expand the therapeutic options for obesity, individuals with obesity will benefit from a diverse range of pharmacological alternatives, reducing reliance on bariatric surgery and a single drug. These additional drugs will also offer relief to patients with type 2 diabetes, who have experienced a year-long shortage of Ozempic due to its prevalent off-label use for obesity.

The availability of effective weight-loss drugs marks a substantial shift in societal perception of the disease and how individuals living with obesity experience it, impacting them in two key ways. First, it promotes the recognition of obesity as a chronic disease, contributing to the destigmatisation of the widespread yet inaccurate notion that obesity solely results from lifestyle choices and a lack of discipline. Second, it empowers patients by offering them the chance to regain control of their health, improve their quality of life, and age more healthily. This will also help to alleviate the mental burden associated with the disease.

Considering the role of obesity in premature mortality from non-communicable diseases (NCDs), the incorporation of these efficacious drugs for both adults and adolescents, alongside public health interventions regulating environmental and commercial determinants of unhealthy eating, will alleviate the burden of this disease. Such progress brings us closer to fulfilling two pivotal targets of the Sustainable Development Goals (SDGs): ending all forms of malnutrition (SDG 2) and reducing premature mortality from NCDs by a third (SDG 3) by the year 2030.

While these drugs do not signal the end of the battle against obesity, they represent a beacon of hope for individuals with obesity representing a milestone in public health strategies aimed at tackling obesity and its associated comorbidities. The integration of therapeutic options for obesity into broader public health initiatives is essential to provide a comprehensive, multisectoral approach in combating the escalating obesity epidemic.

